# A new era of synthetic biology—microbial community design

**DOI:** 10.1093/synbio/ysae011

**Published:** 2024-07-16

**Authors:** Anna Matuszyńska, Oliver Ebenhöh, Matias D Zurbriggen, Daniel C Ducat, Ilka M Axmann

**Affiliations:** Computational Life Science, Department of Biology, RWTH Aachen University, Aachen 52074, Germany; Cluster of Excellence on Plant Sciences, CEPLAS, Heinrich Heine University Düsseldorf, Düsseldorf 40225, Germany; Cluster of Excellence on Plant Sciences, CEPLAS, Heinrich Heine University Düsseldorf, Düsseldorf 40225, Germany; Institute of Quantitative and Theoretical Biology, Heinrich Heine University Düsseldorf, Düsseldorf 40225, Germany; Cluster of Excellence on Plant Sciences, CEPLAS, Heinrich Heine University Düsseldorf, Düsseldorf 40225, Germany; Institute of Synthetic Biology, Heinrich Heine University Düsseldorf, Düsseldorf 40225, Germany; MSU-DOE Plant Research Laboratory, Michigan State University, East Lansing, MI 48824, United States; Department of Biochemistry & Molecular Biology, Michigan State University, East Lansing, MI 48824, United States; Institute for Synthetic Microbiology, Heinrich Heine University Düsseldorf, Düsseldorf 40225, Germany; Cluster of Excellence on Plant Sciences, CEPLAS, Heinrich Heine University Düsseldorf, Düsseldorf 40225, Germany; Institute for Synthetic Microbiology, Heinrich Heine University Düsseldorf, Düsseldorf 40225, Germany

**Keywords:** community design, modular modelling approach, computational biology, synthetic biology, synthetic communities

## Abstract

Synthetic biology conceptualizes biological complexity as a network of biological parts, devices, and systems with predetermined functionalities and has had a revolutionary impact on fundamental and applied research. With the unprecedented ability to synthesize and transfer any DNA and RNA across organisms, the scope of synthetic biology is expanding and being recreated in previously unimaginable ways. The field has matured to a level where highly complex networks, such as artificial communities of synthetic organisms, can be constructed. In parallel, computational biology became an integral part of biological studies, with computational models aiding the unravelling of the escalating complexity and emerging properties of biological phenomena. However, there is still a vast untapped potential for the complete integration of modelling into the synthetic design process, presenting exciting opportunities for scientific advancements. Here, we first highlight the most recent advances in computer-aided design of microbial communities. Next, we propose that such a design can benefit from an organism-free modular modelling approach that places its emphasis on modules of organismal function towards the design of multispecies communities. We argue for a shift in perspective from single organism–centred approaches to emphasizing the functional contributions of organisms within the community. By assembling synthetic biological systems using modular computational models with mathematical descriptions of parts and circuits, we can tailor organisms to fulfil specific functional roles within the community. This approach aligns with synthetic biology strategies and presents exciting possibilities for the design of artificial communities.

**Graphical Abstract**

**Figure:**
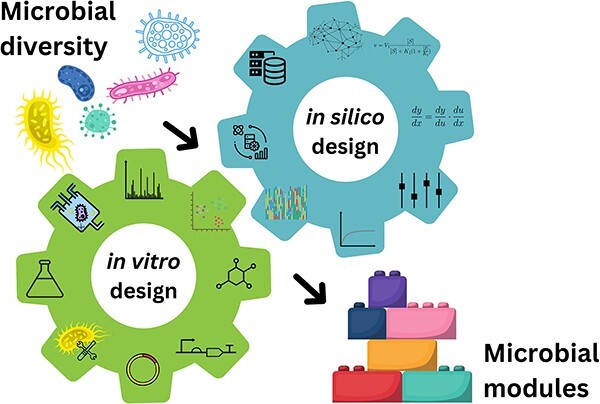

## 1. Introduction

It is increasingly evident that microbial consortia are a ubiquitous feature of most ecological niches and that features of these communities play important roles in a variety of emergent properties at the meso- and macro-scale. Popularized examples of such consortia include the gut microbiome of humans and the soil microbiome associated with plants, both of which play crucial roles in organismal health and disease. In parallel with an increased appreciation of the natural functions of microbial consortia, molecular biology tools have matured in power and precision, giving rise to a nascent interest in the engineering of complex natural communities and the rational design of defined microbial communities from the ‘bottom-up’. Proposed applications for synthetic microbial consortia are broad, including mitigation of human disease [1], increased crop productivity [2], and sustainable production of valuable biomolecules [3, 4]. Yet, there is a significant gap between the theoretical potential of microbial consortia and examples of their successful implementation on real applications on a larger scale, for example, fermentation, raising valid questions about hype versus deliverables. In this Perspective, we explore the idea that key tenants of synthetic biology—especially modularity of biological function—may provide a useful framework with which to conceptualize complex microbial networks and inform the development of new quantitative models. We suggest that abstracting individual species within consortia to ‘modules of community function’ may assist in translating insights gained on context-specific observations of any particular microbial assemblage to design principles useful across different platforms. We discuss how building coarse-grained models based on ‘modules of community function’ could have valuable predictive power to assist future research on microbial community engineering and consider challenges such models may encounter at present.

## 2. From synthetic parts towards communities

Synthetic biology is now a mature field of research with an established record of newly developed biological molecular tools, devices, and cellular structural and functional modules. A core tenet of synthetic biology is that biological systems can exhibit the emergence of highly complex behaviours by virtue of the interconnection between simple ‘modules’ (e.g. DNA regulatory elements, and protein domains) that exhibit robust, well-defined functions regardless of the organismal context in which they are contained. Synthetic biology principles have found relevance and have been applied in the research design for a wide range of topics from fundamental to biotechnological, providing promising solutions to global challenges [5, 6].

The construction of the first synthetic circuits at the ‘dawn’ of synthetic biology was highly reliant on breaking genetic control systems down into definable parts and then collecting and using existing quantitative data that were most important for the function of these parts when used in different contexts [7, 8]. While earlier engineering was, in part, restrained by technical limitations on what could be cloned, this restriction has largely evaporated in recent years. Improved DNA synthesis and assembly techniques have laid the groundwork for designing increasingly complex systems, such as biocellular and cell-free multipurpose metabolic factories, and the design of synthetic protocells and xenobiological systems [9–11]. Currently entering its third decade [12], synthetic biology has achieved a remarkable level of maturity, paving the way for ground-breaking advances in designing increasingly complex systems. Increasingly powerful DNA synthesis and recombination tools also enable the field to consider conceptualizing and manipulating much larger ‘modules’ of function (e.g. multiple genes in a metabolic pathway), or even whole bacterial [13] and eukaryotic cells [14].

One example of recombining large functional modules is the design of multispecies synthetic microbial communities: where individual microbial strains may be of natural origin or genetically engineered to conduct desired functions [15]. Still, many current applications in the literature were achieved by following a more handcrafted and intuitive type of approach, rather than following the whole engineering cycle and utilizing a quantitative approach. Realizing success in this second phase of synthetic biology will require a return to the principles of quantitative characterization and mathematical modelling to improve efficiency and reduce development times. A great example of such an approach is provided by the next-generation individual-based model (IbM) in ecology, where based on the well-known mechanisms of organism interaction, predictive models can be developed to simulate community dynamics [16, 17].

Microorganisms usually coexist in highly complex and dynamic communities, where individual species within the community can occupy distinct roles depending on the population density, environmental conditions, and resource availability. Due to numerous interactions among their members, microbial communities achieve very complex functions. Specific functions exhibited by the whole community (e.g. metabolic transformations) would be unattainable by an individual organism alone. Microbial communities have been successfully used for centuries in some biotechnological processes (e.g. fermentation and waste treatment), despite a lack of formal understanding of individual species or fundamental knowledge of how communities are formed and sustained. Understanding and exploiting these communal capabilities is an emerging field in synthetic biology [18] closely linked to the Microbial Consortia Engineering field [19]. The long-standing use of microbial communities in niche applications provides us with increased confidence in the long-term potential of synthetic microbial consortia—but only if the field can harness the power of the design–build–test–learn cycle to reimagine their applications in a wider range of biotechnologies [20].

**Figure 1.**
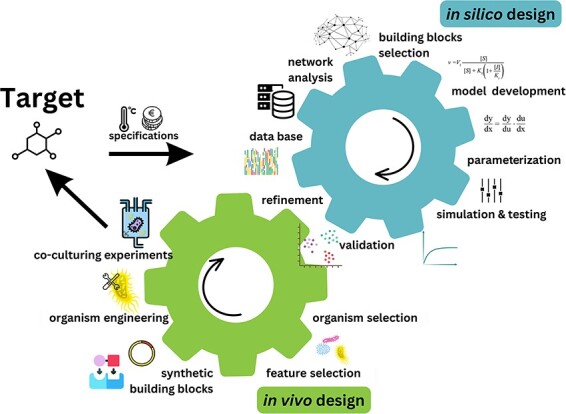
Computer-aided engineering cycles applied to biology. Gears represent the synergy and continuous engagement of computational (*in silico*) and synthetic (*in vivo*) work. A target molecule or synthetic system is created based on specification (e.g. cost and environmental conditions). It is then matched with the database containing information on organismal-producing capacity (measured or derived through computational methods, e.g. constrained-based modelling or network expansion). Based on the information from the database organisms will be selected and then only their key features will be analyzed (e.g. *Ustilago maydis* producing itaconic acid is being reduced to a module that can produce an itaconic acid). Such synthetic building blocks are then translated into mathematical terms from which a dynamic model can be created following the classical approach of modelling (development, calibration/parameterization, testing, validation, refinement, and prediction). Once validated, the model’s output can propose the theoretically best coculturing selection of features and organisms yielding the production of a target molecule or the creation of a stable synthetic community under specified conditions.

How to create a *de novo* synthetic community? We propose a paradigm shift in the way computational models are created to aid artificial community design. Instead of focusing on organisms, we propose to focus on the functional roles that organisms fulfil within a community (see Fig. 1  *in silico* design). This entails that models should be designed to describe the desired functions, independent of which particular microbial species perform the specific function. We propose that ‘when designing a community with a certain purpose, it is irrelevant which specific organisms it contains: organisms are merely the chassis containing necessary metabolic pathways, providing required functional roles’.

## 3. Modelling for synthetic biology

Mathematical and computational biology is becoming an integral part of studies of biological systems across many scales. They offer quantitative methods for rigorous hypothesis testing. Coupled with available large datasets, they organize information into interconnected knowledge hubs and offer guidance for experimental exploration. As the complexity of designs increases, we need to use modelling to guide the first steps in the design phase of the first cycle [21]. To make predictive models, we need a thorough quantitative understanding of the parts and the properties emerging from their interaction to be able to combine them, designing more complex systems. Most recently, great effort has been put into integrating modelling to instruct synthetic biology approaches, in as much the same way as predictive models guide engineers to design new technical devices expanding the range of available tools [22, 23].

### 3.1 Challenges

With modern computational power and parallelization support [24], it is technically no longer a problem to assemble and simulate systems of thousands of equations, yet we face more fundamental difficulties in devising a useful simulation of all processes inside an *Escherichia coli* cell. Whereas a simulation of engine combustion is based on a few, rather simple, equations, describing, for example, continuity, energy, and momentum [25], there are no universal equations describing the single cellular processes. Every enzymatic reaction and every transcription factor-binding process must be described by some equation that needs to be experimentally validated and parameterized with tedious, time-consuming, and usually wet-lab experiments. Unfortunately, the production of new, high-throughput data collections by individual labs will not solve all the challenges that hinder the development of powerful predictive models. For once, we require extensive standardization [26, 27], which has been proven to be key in other engineering fields. However, despite community awareness [28] and excellent, coherent efforts [29], standardization is unfortunately still not technically feasible or not a priority for academic research, nor funding agencies [30]. Additionally, when simulating biological systems one has to face uncertainties on two levels: like in complex physical systems, the complexity alone will bring forth emergent, and intrinsically unpredictable, properties. In addition, the single entities that make up the biological system are only approximately characterized and often their behaviour is not fully explained by an underlying theory.

Until a more fundamental understanding of the organizational principles of living systems is established, we must deal with the dilemma of highly complex systems that lack solid theoretical foundations, leaving us with a limited number of practical solutions to navigate trade-offs between controllability and complexity [31]. One current option is to reduce the complexity of our models, so that we can understand and systematically test all assumptions made while constructing the model. In this way, we will gain control, and thus understanding, of the system under investigation at the expense of a detailed quantitative predictability. Such simple models are extremely useful and have also been successfully employed to design synthetic systems (see genetic toggle switch [7]). Alternatively, we can assemble highly complex and extremely detailed models and feed these with the enormous amount of experimental data available, copying the digital twin (DT) approach. In industry, DTs are virtual accurate representations of physical objects assembled from numerous digital components, providing cost-effective alternatives to physical objects [30, 32]. This approach trades control and understanding in favour of predictability. Conceptually, they follow the machine learning and artificial intelligence approaches, by which highly complex models are automatically constructed by algorithms which reproduce the data very well. Although they prove to be extremely useful in making quantitative predictions and are currently developed for many biofoundries [33], due to their complexity and specificity, it is nearly impossible to infer common and universal principles that will hold true for other biological systems. Hence a promising alternative emerges from the combination of mechanistic understanding with machine learning algorithms in the form of physically constrained recurrent neural networks. This new generation of models has been recently trained to predict microbial community dynamics and target functions, and was used to select experimental conditions to maximize desired metabolite production with the minimal set of experiments leading to the optimal set-up [34]. Although as a neural network-based model, this approach provides a limited opportunity to extract new knowledge about the system.

### 3.2 Organism-free modular approach towards synthetic community modelling

Despite the challenges mentioned earlier, the computational design of a stable, complete synthetic two [17] and three-strain microbial consortium has been recently successfully completed [35]. It is also important to note that there already exist numerous tools to support the *in silico* design of new pathways, and new toolkits for synthetic design are developed based on the results from ensemble dynamic modelling approach [4]. This highlights the significant progress made in this field and the promising potential for further advancements [33]. Many mathematical approaches that proved to be successful in studying the metabolism of a single organism have been adapted to study consortia dynamics. In particular, constraint-based modelling techniques on genome-scale models, such as flux balance analysis (FBA) are successfully applied to consortia of well-studied organisms (see a recent review of classical methods and available tools [36]). Several open-source packages to study communities were developed, e.g. Microbiome Modeling Toolbox [37] or MICOM, encapsulating optimization techniques specifically for studying gut microbiota [38]. Moreover, networks exploring the dynamic FBA (dFBA) have been developed and successfully applied to study the emergent properties of subpopulations of a monoculture [39], predict the optimal partner to form a consortium via pairing experiments [40] or explain steady-state population distributions of a three-species consortium [41], with COMETS being the most recent and comprehensive one, with broad applicability to microbial communities [42]. However, despite the knowledge of microbial interactions that these methods provide, they do not yet follow a rational approach for a new design of an artificial organism within an artificial community. Whether it is a production of a completely novel compound or just a more efficient production of a known one, computational models should inform the choice of molecular modules, e.g. by providing quantitative predictions regarding the performance of the end product. Ideally, computational frameworks could guide as in the work by Schwander *et al*., where using theoretical tools and simple laws of thermodynamics, new enzymatic steps have been proposed to be included in the carbon fixation pathway, guiding the creation of a more efficient, synthetic CO_2_ fixing pathway [43].

We argue, that to unlock the full potential of synthetic biology (e.g. to develop new antimicrobial compounds, to engineer more efficient synthesis pathways), it is necessary to move from the well-known model organisms into uncharted territories of non-model organisms, for which quantitative data are sparse. In fact, we see a possibility of abandoning the organism-centred perspective completely and instead focusing on the functions that organisms provide to the community. As in a recent example, evolutionary game theory can be used to predict the dynamics of a two-species community within different environmental landscapes by considering their trade-off and costs depending on their chosen strategies [44]. Several computational approaches allowing this shift have been recently proposed. On the one hand, with new tools such as μbialSim, interpretations of the experimental meta-OMICS data focusing on metabolite exchange as the main interaction type allow the simulation of hundreds of species of the gut microbiome [45]. This method, although promising, is limited by the availability of huge data collections. Therefore, another possible direction is to follow a modular approach, where complex metabolic networks are simplified to more manageable modules, based on their functional role [46, 47]. Typical functions which are important for the construction of communities may include the production of certain metabolites, provision of nutrients, and exhibiting protective functions for other community members, such as through the production of antibacterial compounds. These modules can then be stored in a database, much like BioBricks [48] or the Registry of Standard Biological Parts [49, 50], to provide a library of circuits to choose from. Such a coherent database with modules described in a standardized format could facilitate the automatic creation of artificial communities. Recently, several examples of a computer-aid community design emerged, including software development. McComedy (Microbial Communities, Metabolism, and Dynamics) is a platform that allows IbM creation of microbial systems, based on their consumer–resources relationship. Simple building blocks (‘process modules’) can be combined and parametrized based on the specific research question [16]. Karkaria *et al*. [35] have, on the other hand, used the assumption about the bacteria’s ‘capacity to produce’ antimicrobial peptides (bacteriocins) to identify the most appropriate candidates (models), which in combination leads to a stable two or three-strain community. Finally, Sakkos *et al*. [17] recently used IbMs to predict the regulation of consortia growth depending on sucrose productivity.

In Fig. 1, we provide a schematic of such an organism-free modular process of computational modelling, supporting the synthetic design. The starting point for the construction of a community with a desired behaviour is a database or a library of modules describing common biological motives. These modules can range in complexity from simple molecular cycles resulting in protein modifications, like phosphorylation/dephosphorylation, which is a primary mechanism for signal transduction in cells, to more complex subsystems, such as whole metabolic pathways. These modules should be described by dynamic mathematical models that can quantitatively simulate changes in their functional role. The behaviour in turn is specified by the requirements desired by the synthetic biologist. Such specifications may consider the abundance of a given metabolite, the dynamic behaviour of a community (e.g. oscillation), competitive interaction between the community members, etc. Hence, we use two interlocking gears as a visual representation of the *in silico*’s work capacity to impart input to subsequent cycles. The proposed modular design allows for combining the modules in a flexible manner and studying the emergent properties of a large number of complex networks consisting of different combinations of modules. In particular, such models will provide a framework to systematically study whether the behaviour of isolated parts is preserved when assembled into larger circuits and therefore will be useful for further studies of such phenomena like metabolic division of labour, where a metabolic task is divided into complementary steps across different interacting strains [51]. In this way, artificial community networks can be designed in a predictive manner by assembling different modules that best match the specification requirements.

It could be argued that moving towards models that are species-independent may be an oversimplification of necessary biological features that are inherently complex. It is obvious that the abstraction of organisms as modules of function does, by definition, remove some details from a consortia model, yet prior art and newly emerging datasets provide support that this approach can have value. By analogy, ecological models at the meso- to macro-scale have now exhibited considerable predictive capabilities for decades without needing to account for the myriad ways in which organisms may subtly differ (e.g. different species of grasses or pollinators [52]). Similarly, there are an increasing number of examples that specific species composition of complex microbial communities may change over time/geography, and specific community functionalities are stable on a broader scale. As an illustrative example, a recent metagenomic survey of heterotrophic bacteria associated with the photosynthetic diatom, *Phaeodactylum tricornutum*, identified three functional ‘guilds’ of microbial activity (i.e. macromolecule remineralizers, macromolecule users, and small-molecule users) that were constant across different communities but which lacked phylogenetic relationships [53].

## 4. A new era of synthetic biology—community design

Microbial communities exhibit many advantages over monocultures due to division of labour, spatial organization, and robustness to perturbations. Communities have unique catalytic activities and can use complex substrates by compartmentalization of pathways and distribution of molecular burden. We now have the synthetic biology tools and approaches at hand that allow us to construct and control communities by engineering their communication, gene expression, and metabolic interactions [23]. Controlling the behaviour of individual species within a community has consequences for population dynamics. Computational models of metabolism and population dynamics, which can predict metabolic cross-feeding and infer population dynamics over time, help us to resolve complexity and systematically engineer communities [54, 55]. Combining model-based hypotheses with omic studies helped in the design and implementation of synthetic phototrophic microbial communities [18, 56].

To fully unlock the creative potential of synthetic biology toward the challenge of *de novo* design of complex biosystems, like synthetic microbial communities, three main research avenues need to be further developed. (i) On the biological side, we need a wide range of methods and technologies for obtaining quantitative data on the parts to be used and on already previously assembled devices (databases). (ii) On the modelling aspect, we require improvements in the predictive power of the already available, first *ad hoc*, mostly ordinary differential equation models for simple networks, to be finally able to use them to guide the assembly of more complex molecular and cellular metabolic, signalling, and community behaviour networks. (iii) Finally, tight collaborations between experimentalists and theoreticians should become standard. We believe that this should start by teaching the new generations of biologists more quantitative methods and a quantitative approach towards biological systems; so eventually, we can abandon the distinction between ‘classic’ and ‘computational’ biologists [57].

However, detailed mechanistic insights into microbe–microbe interactions often remain undetermined. Although online monitoring systems and diverse biosensors are available for microbial cultivation [58], quantitative and dynamic data are very limited for most microbial communities. Understanding the basic principles of metabolic exchanges is essential to control the intercellular interactions, spatiotemporal coordination, robustness, and stability of synthetic microbial communities. A key challenge for designing stable interactions within synthetic consortia is to understand how functional roles can be partitioned across a microbial community in productive compartments to achieve desirable population-level behaviours. How to define such a partition or a universal function? Here, we see the potential in organism-free modular modelling approaches to guide complex community design and to instruct building synthetic communities for exploiting novel behaviours and applications in biotechnology, medicine, and agriculture. The key lies in focusing on metabolic interaction and functionality rather than on an organism itself. Computer-aid simulations will help to narrow down the possible targets and answer the question: ‘What components are necessary to encode a specific behaviour within the community?’ This, paired with collecting well-thought ‘high-level’ quantitative data, will fully unlock the potential of computer-aid community design.

## Data Availability

No data were collected in this work.
